# Clinical Outcome Assessment in Cancer Rehabilitation and the Central Role of Patient-Reported Outcomes

**DOI:** 10.3390/cancers14010084

**Published:** 2021-12-24

**Authors:** Jens Lehmann, Maria Rothmund, David Riedl, Gerhard Rumpold, Vincent Grote, Michael J. Fischer, Bernhard Holzner

**Affiliations:** 1 Department of Psychiatry, Psychotherapy, Psychosomatics and Medical Psychology, University Hospital of Psychiatry II, Medical University of Innsbruck, 6020 Innsbruck, Austria; maria-sophie.rothmund@i-med.ac.at (M.R.); david.riedl@i-med.ac.at (D.R.); gerhard.rumpold@tirol-kliniken.at (G.R.); bernhard.holzner@tirol-kliniken.at (B.H.); 2 Ludwig Boltzmann Institute for Rehabilitation Research, 1140 Vienna, Austria; vincent.grote@rehabilitation.lbg.ac.at (V.G.); michael.fischer@reha-kitz.at (M.J.F.); 3 Vamed Rehabilitation Center Kitzbühel, 6370 Tyrol, Austria

**Keywords:** outcome assessment, patient-reported outcomes, patient reported outcome measures, rehabilitation, cancer rehabilitation, value-based care

## Abstract

**Simple Summary:**

After completion of acute cancer treatment, it is important to support patients in recovering physically and psychologically and to help them regain their social life. This is the goal of cancer rehabilitation. If we want to know which rehabilitation interventions are helpful, we must measure their effects. This can be done by asking clinicians, testing patients’ performance, observing their behaviors, or by asking patients directly about their experience. This paper focuses on reports from the patients. We give an overview of available questionnaires and offer advice regarding their use. Furthermore, we discuss how to integrate them into clinical practice and research. The most promising way to collect such data are electronic systems, which offer many advantages. The goal of assessing the patient perspective is to help patients, clinicians, and health insurance providers to decide which rehabilitation interventions suit patients’ needs, and therefore, which ones should be chosen and reimbursed.

**Abstract:**

The aim of cancer rehabilitation is to help patients regain functioning and social participation. In order to evaluate and optimize rehabilitation, it is important to measure its outcomes in a structured way. In this article, we review the different types of clinical outcome assessments (COAs), including Clinician-Reported Outcomes (ClinROs), Observer-Reported Outcomes (ObsROs), Performance Outcomes (PerfOs), and Patient-Reported Outcomes (PROs). A special focus is placed on PROs, which are commonly defined as any direct report from the patient about their health condition without any interpretation by a third party. We provide a narrative review of available PRO measures (PROMs) for relevant outcomes, discuss the current state of PRO implementation in cancer rehabilitation, and highlight trends that use PROs to benchmark value-based care. Furthermore, we provide examples of PRO usage, highlight the benefits of electronic PRO (ePRO) collection, and offer advice on how to select, implement, and integrate PROs into the cancer rehabilitation setting to maximize efficiency.

## 1. Introduction

An increasing number of people are diagnosed with cancer each year [1]. Due to improved diagnostics and treatment during the last few decades [2], survival rates are also rising [1,3]. Thus, healthcare systems are facing an increasing number of cancer survivors, many of whom suffer from various short- and long-term effects of their disease and/or treatment [4,5]. For the term “survivor”, different definitions exist [6]. In the present review, the term “survivors” is used to refer to anyone who has completed acute treatment, is currently recovering from treatment, and is, thus, potentially receiving any kind of post-acute rehabilitation care. To minimize patients’ impairment after completion of acute treatments, it is crucial to provide options for rehabilitation and long-term care.

Rehabilitation is defined as “a set of interventions designed to optimize functioning and reduce disability in individuals with health conditions, in interaction with their environment” [7]. Cancer rehabilitation is recommended in numerous clinical guidelines and can comprise diverse interventions, including physical therapy and activity (e.g., exercises, yoga, lymphatic drainage, etc.), supportive medications (e.g., for pain or insomnia), psychological interventions (e.g., resiliency training, coping strategies, relaxation techniques, etc.), and assistance for social (re)integration (e.g., preparing to return to work) [8]. Such interventions can be offered in the form of in-patient care in rehabilitation clinics or provided as ambulatory or home care for out-patients (see [9] for a comprehensive definition).

Regardless of the context and type, the effectiveness of interventions must be evaluated in order to judge their benefits for patients and justify their usage. Little research has been done on the key factors involved in rehabilitation treatment programs. While research on anti-cancer treatments focuses primarily on survival or tumor response [10], rehabilitation “aims to maximize a person’s ability to live, work and learn” [7]. Thus, the outcome of interest in rehabilitation research and evaluation is patients’ functioning and health-related quality of life (HRQOL). To assess such outcomes, different types of clinical outcome assessments (COAs) are available, ranging from clinician or proxy ratings, to measuring patients’ performance on predefined tasks, and finally to patients’ self-reporting. With increasing efforts to provide patient-centered research and treatments, patient-reported outcomes (PROs), in particular, are gaining in importance.

In the present narrative review, we will discuss the benefits and drawbacks of different types of COAs and how they can complement each other. Moreover, we place a special focus on PROs, which assess and evaluate cancer rehabilitation from the patient perspective.

## 2. Outcome Assessments in Cancer Rehabilitation

### 2.1. Functioning, Disability, Health, and Quality of Life as Outcomes of Interest

Before discussing ways of assessing COAs, the outcomes of interest are briefly defined. In rehabilitation settings, the goal is to restore and/or improve patient functioning and quality of life. Both concepts seem to be intuitive at first glance, but are difficult to define precisely and, thus, are often used interchangeably [11,12].

**Figure 1:**
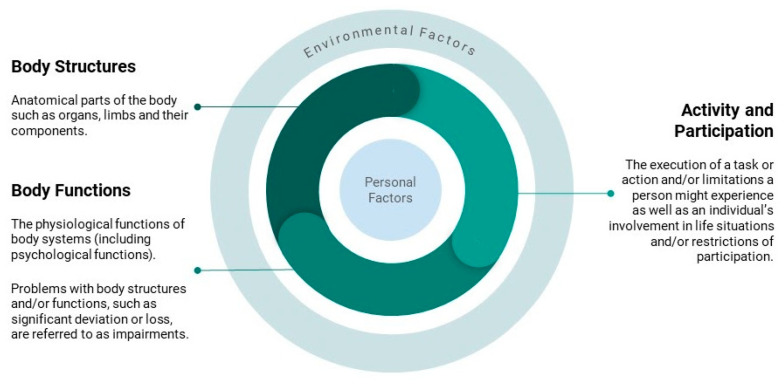
Illustration of the International Classification of Functioning (ICF).

Perhaps the most comprehensive terminology to describe such outcomes is the International Classification of Functioning, Disability, and Health (ICF) by the World Health Organization (WHO) [13]. In contrast to the International Classification of Diseases (ICD), the ICF does not define criteria for diagnostic decision-making based on the presence or absence of a disease or disorder. Instead, functioning and disability are seen as a continuum: “Functioning is an umbrella term for body functions, body structures, activities and participation. It denotes the positive aspects of the interaction between an individual (with a health condition) and that individual’s contextual factors (environmental and personal factors)”. Ref. [13] Disability, on the other hand, refers to the negative aspects of the same components, which are illustrated in Figure 1.

This basic model of functioning covers the major domains of health: body structures and functions subsume physical, but also psychological (i.e., mental health and cognitive issues) aspects; social health is represented in activity and participation. All these aspects of health are influenced by environmental (e.g., work and family) and personal (e.g., sex, age, and ethnicity) factors.

These domains are also fundamental to the definition of HRQOL. Nevertheless, health status and quality of life (QOL) can be differentiated from one another. While functioning, disability, and health are generally understood as *objective* concepts in the ICF terminology, HRQOL refers to the *subjective* experience of these issues [11]. The WHO defines HRQOL as an “*individual’s perception* [emphasis our own] of their position in life...incorporating in a complex way individuals’ physical health, psychological state, level of independence, social relationships, personal beliefs and their relationships to salient features” [14].

### 2.2. Different Types of Clinical Outcome Assessments (COAs)

**Figure 2:**
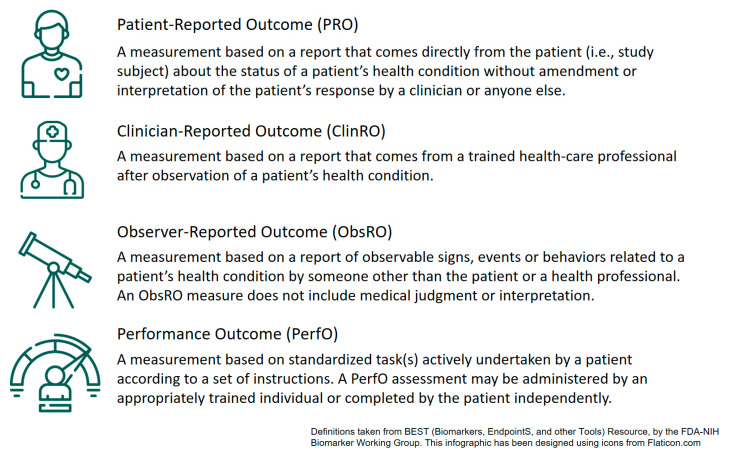
Different types of clinical outcome assessments (COAs).

There are different ways to assess these clinical outcomes (see Figure 2), each with different strengths and limitations. The first type of COAs are clinician-reported outcomes (ClinROs). ClinROs are any ratings made by clinicians from their expert perspective. These ratings can be based on evaluations of objective biomarkers (e.g., blood count), but also on observable behaviors of the patient. ClinROs, therefore, comprise the documentation of evaluations of a patient’s health status by healthcare providers; however, they are seldom documented in a structured and consistent manner. Well-known examples of structured ClinROs are the Eastern Cooperative Oncology Group (ECOG) status [15] or the Karnofsky Index [16], which grade a patient’s overall health status in very basic stages from death to full function and activity.

Based on observational behaviors, not only clinicians, but also parents, partners, or other people related to a patient can report a patient’s health status (ObsROs). However, while this plays a central role in pediatric settings or patients with cognitive impairments [17,18], ObsROs are less common in adult cancer rehabilitation.

ClinROs and ObsROs are based on patients’ daily observable behaviors, which can vary greatly and which may be perceived differently by different raters. To have a more reliable and comparable basis for evaluation, performance-based outcomes (PerfOs) can be assessed. To do so, patients are asked to complete standardized tasks. This enables the measurement of outcomes by objective and quantifiable means; for example, the time needed to complete a task, the number of errors made, or other objective parameters, such as muscle strength or stamina. However, variability in such measures may still be influenced, for example by patients’ motivation or the setting they are conducted in.

Commonly used PerfOs are, for instance, the Timed Up and Go (TUG) Test [19], measuring the time it takes for patients to stand up from a chair, walk a specific distance, and sit down again, or the Hand Grip Strength Test [20], measuring strength with a dynamometer. Besides physical parameters, cognitive functions such as attention, memory, problem-solving, or specific verbal or visuospatial skills can be assessed using PerfOs [21].

As they are based on standardized and specific tasks, single PerfOs cannot comprehensively assess functioning or QOL [22], but only very distinct abilities. Nevertheless, research has shown that various PerfOs, such as gait speed, hand-grip, and verbal memory, can predict survival [20,23], indicating that their informative value extends beyond the specific tasks completed.

After all, neither ClinROs/ObsROs nor PerfOs can give insight into unobservable aspects, such as emotions or fatigue. Mental health, as well as patients’ subjective perception of their own functioning, are fundamental components of QOL. Therefore, it is crucial to assess PROs as well. A PRO is defined as “a report that comes directly from the patient (i.e., study subject) about the status of a patient’s health condition without amendment or interpretation of the patient’s response by a clinician or anyone else” [24,25].

Following this definition, PROs are not only crucial for the assessment of psychological well-being or internal issues, but they should also be used for any outcome that the patient can sensibly self-report (guidance by FDA/EMA, [25,26]). This includes observable functions that might also be assessed with ClinROs, ObsROs, or PerfOs. Research has shown considerable discrepancies in ratings depending on the perspective and type of COA. For example, the correlation between ClinROs and PROs varies depending on the symptoms that are assessed: correlations are higher for observable symptoms (e.g., vomiting or diarrhea) and lower for inherent symptoms (e.g., fatigue or pain) [27]. In clinical trials, ClinROs are less sensitive for detecting changes in adverse events compared to PROs [28]. A systematic review of the associations between ClinROs for functional performance status assessments and PROs for the same domain found that the association was “moderate at best”, which indicates that both PROs and ClinROs offer a unique informational value [29]. The same is true for the level of functioning, for which PerfOs can be used to obtain objective measurements of task completion abilities. Nonetheless, PerfOs show rather low correlation with PROs in functional domains, indicating that timed tests cannot measure all aspects of functioning and complementary COAs are needed [30,31].

Another example highlighting the need for a combination of different COA types is work ability. As cancer is associated with unemployment [32], one major interest of rehabilitation and service providers is the (re)integration of survivors into work, making the percentage of patients working after rehabilitation an important indicator of success. However, the observable factor, i.e., whether a person has a workplace or not (ObsRO), does not really provide information on their performance or satisfaction at work, which can only be assessed using PerfOs [33,34] or PROs [35].

To conclude, a combination of different COAs is needed for the comprehensive evaluation of patient needs, and the effectiveness of different cancer rehabilitation programs: ClinROs or ObsROs, which are considered good indicators for functioning and disability [36], PerfOs, which provide more objective parameters, and PROs, which take the patient perspective into account. Only the latter are able to assess internal issues that can only be reported by patients themselves, ranging from emotions and mental health to the subjective experience of functioning, disability, health, and QOL. The next chapter will provide an overview of the types of patient-reported outcome measures (PROMs) that can be used in cancer rehabilitation.

## 3. PROMs for Cancer Rehabilitation

There is a wide range of instruments available for assessing PROs. A common approach for classifying these so-called PROMs is to classify the contents that are assessed. Multidimensional instruments assess overall quality of life by covering physical, psychological, and social aspects, as for instance the EORTC QLQ-C30 [37]. Other PROMs focus on single outcomes or domains. Among the most commonly used PROMs for psychological distress are the Hospital Anxiety and Depression Scale (HADS, [38]) and the Beck’s Depression Inventory (BDI-II, [39]). Another example of a domain-focused PROM is the Work Ability Index (WAI, [40,41]), which can be used to measure a patient’s perception of their working abilities.

A further relevant domain is patients’ satisfaction with care. Even though only assessable by patient perspective, this aspect is somewhat different from PROMs assessing patients’ health condition. Thus, a distinction is made between PROMs and so-called patient-reported experience measures (PREMs) [42]. While many PREMs do not adequately cover all important aspects of care [43], the EORTC Satisfaction with Cancer Care core questionnaire (QLQ-PATSAT-C33) and out-patient module (QLQ-OUT-PATSAT7) [44] can be used to assess the satisfaction with care specifically in patients with cancer.

There are also a number of different measurement inventories or item banks available that offer various scales, short forms, or items focusing on single domains, which can be combined for multidimensional assessments. Among those are the EORTC Item Library [45], the Patient-Reported Outcomes version of the Common Terminology Criteria for Adverse Events (PRO-CTCAE) library [46], and the Patient-Reported Outcomes Measurement Information System (PROMIS©, [47]).

Another way of looking at PROMs is to compare generic and specific PROMs. Generic PROMs can be used in all kinds of populations, thus allowing comparisons across different groups and diseases. Some examples of commonly used generic PROMs in cancer rehabilitation are the EQ-5D [48,49] and the SF-36 [50]. In contrast, specific PROMs are tailored to a certain disease or patient population. For example, the EORTC QLQ-C30 or the FACT-G [51] are questionnaires for patients with cancer. Due to their specific nature, they can more accurately assess issues that are peculiar to the target population and, thus, offer superior measurement validity. At the same time, however, they do not offer the same benefit of wide comparability as generic instruments. When choosing between a generic and a specific instrument, it is important to remember that those characteristics lie on a continuum. For example, the EORTC QLQ-C30 is specific to patients with cancer without being limited to a distinct cancer entity; instead, it can be complemented with a range of more specific modules for different cancer types, sites, or stages.

PROMs can not only be specific to certain diseases or diagnoses, but can also be specifically designed for different age groups, which have different concerns and cognitive abilities. For example, children might need simpler questions and a less complex response scale in order to provide valid and reliable self-reporting [52]. Adolescents and young adults, on the other hand, can properly complete adult tools [53], but face challenges which are specific to their situation in life at the point of transition from childhood to adulthood [54,55]. Thus, several instruments specifically for pediatric settings are currently available [56,57,58] and further tools are also being developed [59].

Finally, there are also PROMs specific for use in certain care settings and contexts. An example of a rehabilitation-specific PROM is the Activity Measure for Post-Acute Care (AM-PAC, [60]). The AM-PAC is a measurement system based on the ICF framework. It assesses physical, personal/instrumental, and cognitive functioning. It can, for example, be used to screen for patients who require special support during rehabilitation. Although the AM-PAC is not cancer-specific, other PROMs have been developed specifically for cancer rehabilitation. An example is the tool by the Cancer Rehabilitation Medicine Metrics Consortium (CRMMC) measuring physical and social function as well as fatigue [61]. Another instrument focusing specifically on the needs and quality of life of patients with cancer is the Cancer Rehabilitation Evaluation System (CARES), which also exists as a short form (CARES-SF) [62]. However, although these measures performed well across different cancer sites and phases of the disease [63], they are either relatively new or have not yet found widespread use [64].

Taking a broader perspective, a review by Duijts et al. [65] showed that to measure HRQOL after the end of primary care, either generic PROMs, such as the SF-36 measure, or cancer-specific PROMs, such as the EORTC QLQ-C30 or FACIT measures, were used. In their review of clinical trials on HRQOL after behavioral or physical interventions for patients with breast cancer, only 1 out of 21 studies used a rehabilitation-specific PROM. One potential drawback of the broad use of cancer-specific PROMs such as the EORTC QLQ-C30 or FACIT measures is that they may not be appropriate for disease-free cancer survivors in their entirety: As argued by van Leeuwen et al., these types of PROMs comprise items assessing acute or treatment-related symptoms (such as vomiting), which are often of lower relevance once treatment has been completed [66]. Conversely, other domains that might be of higher relevance are not covered (fear of recurrence or the ability to return to work). There are a number of survivor-specific PROMs addressing such issues and these PROMs may also be suitable for patients in cancer rehabilitation [67,68,69,70]. However, these PROMs seldom assess the physical long-term effects of cancer or its therapy, and their psychometric validation is limited [71]. The EORTC has recently completed the initial validation of a new set of survivorship questionnaires that are promising for usage in the cancer rehabilitation setting [66,72].

## 4. PROM Usage in Cancer Rehabilitation Research

Historically, the quality of studies in cancer rehabilitation was judged as “less than optimal” in a review of randomized controlled trials covering cancer rehabilitation between 1990 and 2011 [73]. The review found that assessment tools (i.e., mostly PROMs) for functional impairment were often described insufficiently and would benefit from improvement and standardization (see our chapter ‘Recommendations for PROM Selection’). Notwithstanding, there is an increasing body of literature demonstrating how the effects of cancer rehabilitation can be measured using PROMs. A systematic review by Mewes et al. reported that multidimensional rehabilitation interventions showed heterogeneous effect sizes on quality of life in the range of Cohen’s *d* −0.12 to 0.98 [74]. Moreover, the cost effectiveness of interventions in the review was largely positive and ranged up to €16,976 in cost savings per quality-adjusted life-year (a metric of disease burden, which includes the quantity and quality of lived time). However, Mewes et al. also noted that evidence, in general, is scarce and dominated by breast cancer studies, which hinders generalizations across cancer entities. Another systematic (Cochrane) review by Mishra et al. evaluated the effects of exercise interventions on the HRQOL of post-treatment cancer survivors across 40 trials [75]. Exercise interventions are a common part of rehabilitation programs. The review found that there is substantial evidence for the positive effects of exercise interventions on different HRQOL domains. However, the review also notes the heterogeneity of measures used and the risk of bias in many trials.

One of the most comprehensive assessments of HRQOL encompassing different cancer entities has been published by Licht et al. [76,77]. They were able to show the effects of cancer rehabilitation across different cancer entities and treatment modalities in a large single-center study (*N* = 4401). They administered the EORTC QLQ-C30 (symptoms and functional health), the HADS (anxiety and depression), and the WAI (self-rated work ability) instruments before and at the end of cancer rehabilitation. For all measured domains, a significant improvement was found and all effects sizes for gains in functioning were medium to large. Variation across cancer entities was also observed, indicating that different diagnoses have different needs during rehabilitation, which should be accounted for.

An interesting approach for using PROMs to measure the effects of cancer rehabilitation while considering individual needs on the patient level was demonstrated in a study by Nottelman et al. [78]. In their study, the researchers evaluated the effects of an early, integrated, and palliative intervention compared to usual care for patients with advanced cancer. At baseline, patients completed the EORTC QLQ-C30 and chose a “primary problem” from the questionnaire, which was then analyzed as the primary outcome in the study. Compared to care as usual, patients in the intervention arm showed significantly higher improvements regarding their “primary problem” domain and more patients reported having received help with that domain. These, or similar, approaches for identifying individual domains that patients consider important may help future studies select more meaningful outcomes.

In summary, there are different ways in which PROMs can be used in cancer rehabilitation research. Besides health status, they allow the evaluation of interventions from the patient perspective and can be used to measure improvements in functioning domains or symptom burden. However, much of the past research was conducted on samples composed of mainly breast cancer survivors [65,74,79] and validated and psychometrically sound PROMs are often not consistently used [80]. This hinders cross-institutional and generalizable research, which could otherwise serve as a basis for value-based care.

## 5. Moving to PROs to Benchmark Value-Based Care

If the aim is to provide patient-centered rehabilitation care, its evaluation from the patient perspective should be encouraged and acknowledged. PROs can help to assure quality of services across providers and can possibly even be used in decisions on financial reimbursement. PROs may, therefore, contribute to attributing “value” to care. Within the healthcare setting, value can be defined as health outcomes per money spent [81]. However, traditionally, PROs are not included in any of the aforementioned ways in cancer rehabilitation. Instead, patient-centered evaluation of rehabilitation is scarce [82] and reimbursement is mostly provided on a Fee-For-Service basis, where the number or extent of services, and not their impact on patient health, is the determining factor to determine value.

There are many good reasons to use PROs to evaluate rehabilitation from the patient perspective. First and foremost, patients themselves should be able to attribute value to their care or components of care, as they are on the receiving end of it [81]. Including patients’ perspective allows clinicians or researchers to answer fundamental questions about care, such as “How much better do patients like me feel with this treatment?” [83]. Gathering comparative data on the provider level can close such knowledge gaps and allow a meta perspective on the evaluation of cancer rehabilitation. Moreover, PROs have been shown to be associated with other desirable rehabilitation outcomes, such as return-to-work: in a study by Nübling et al., patients who reported to have profited more from rehabilitation also had a better return-to-work rate in the subsequent months and years [84]. There is a clear need for common and mandatory routine data collection procedures with standardized PROMs that can be used for analysis and reporting to improve outcomes and sustainability [85]. They allow assessment within individuals and provide a normative basis for comparing different treatment pathways and environments. Standardization and provision of stratified normative values can make an important contribution to more personalized medicine and to identify critical success factors and non-responders early in the rehabilitation process [86,87]. This highlights how PROs can be used as prognostic factors for rehabilitation success and shows their significance for value-based care.

Some PROMs allow the calculation of health utilities, which have particular importance for value-based care and economic analyses of rehabilitation. Health utilities are a measure of preference or value that patients or society assign to specific health states (i.e., a rating of how desirable a certain health state is). They typically range from 1 (a state of perfect health) to 0 (equivalent to being dead), but occasionally values below 0 are possible, indicating a health state that is less desirable than being dead [88]. Health utilities convey important information about the preferences of health states and allow users to balance the QOL with the length of life, creating measures, such as quality-adjusted life-years. These, in turn, can be integrated into health economic analyses and set in relation to, for example, treatment costs. However, only specific health utility measures allow for the calculation of health utilities. For example, the EQ-5D [48,49] (a generic instrument assessing five general dimensions of health)—although widely used, does not cover all domains that are relevant to patients with cancer. The EORTC Quality of Life Utility-Core 10 Dimensions (EORTC QLU-C10D), in contrast, is a cancer-specific utility instrument for which health utilities can be calculated [89,90,91]; it covers 10 domains, which are particular to the HRQOL of patients with cancer. As it is based on the EORTC QLQ-C30, it allows for cost-utility analyses to be conducted using patients’ HRQOL ratings given in the questionnaire.

The growing importance of PROs has resulted in rising demands by regulators and payers for the patient-centered evaluation of rehabilitation. While this is, on a provider level, still scarce, there are some first examples that show how the patient perspective and patient experience can be integrated into systematic outcome evaluation. For example, satisfaction with rehabilitation and patient-reported effectiveness of the program are now included alongside other factors in a quality assurance index by the German pension fund [92]. Participating centers regularly assess PROs from a randomly selected subset of their patients. These PROs are then, alongside other COAs and benchmarks, included in a central quality assurance index. Such systematic collection of PRO data allows regulators and payers to benchmark different providers and centers against each other and identify those centers and programs that measurably increase patient HRQOL. In many other countries, medical quality outcomes from PROMs or patient satisfaction data are still not public and not accessible to healthcare professionals or patients [85]. Ideally, insurance providers should make these results available, as they also determine and review the structures, processes and content of the healthcare system. At the same time, caution is required as to not selectively include only patients from certain groups or from whom good outcomes are expected and thereby bias outcomes; this may introduce the need for case-mix adjustments [93]. Currently, however, a physician, therapist or patient can only make their expectations and assessments of treatment success based on their personal experience and knowledge.

Another approach toward value-based care is the inclusion of PROs in reimbursement or payment models in healthcare, which is increasingly called for [94]. Especially in North America, there are ongoing debates on how PROs can be made a part of payment models for oncological care. As one of the first adopters, the Oncology Care First model comprises, among other requirements, the systematic assessment of PROs for symptom monitoring [83,95]. Such payment reforms, for now, mainly focus on the implementation of PROs during cancer treatment. However, reforms will likely expand to more elements of cancer care and the adoption of PROs in cancer rehabilitation is likely to become a reality [80].

## 6. Standardized Outcome Sets and Recommendations for PROM Selection

To inform decision-making in healthcare, PROMs and COAs in general must meet fundamental psychometric properties. There is broad consensus on the importance of the validity, reliability, and responsiveness of PROMs and the interpretability of the resulting scores [96]. This is not only a methodological issue, but also implies an ethical responsibility. PROMs should be comprehensive enough to assess all important outcomes, but at the same time, the burden for patients to complete these instruments should be minimal [97]. Consequently, PROMs should only assess outcomes which are actually relevant to the target population and the research question, whereas redundancy between different items, scales, or instruments should be avoided in order to keep assessments feasible.

If one wants to investigate whether there is an improvement over time, it is important to use a consistent set of outcome measures throughout all time points. This aspect is especially difficult in rehabilitation, because patients may often receive care in different institutions at the transition from in-patient care to out-patient rehabilitation care in ambulatory settings or home-based care programs. Therefore, attempts have been undertaken to standardize outcome assessments by defining core outcome sets (COS), i.e., minimal standard sets of outcomes that should be assessed for different purposes.

In a study involving cancer survivors as well as scientific experts and healthcare professionals, Ramsey et al. [98] defined a minimal standard set of PROs which should be assessed in research on cancer survivorship. They identified a list of relevant health and QOL components, including emotional distress (depression, anxiety, and fear of recurrence/progression), available coping strategies, physical symptoms (pain and fatigue), cognitive and physical functioning, role functioning, and the financial toxicity of cancer. They further suggest assessing the overall burden of side effects, overall health status, and overall QOL.

However, in a review of several cancer-specific COS, the same research group found that “Efforts to standardise outcome assessment via the development of COS may be undermined by a lack of recommendations on how to measure core PROs”, and that “To optimise COS usefulness and adoption, valid and reliable instruments for the assessment of core PROs should be recommended” [99]. The COSMIN Group (COnsensus-based Standards for the selection of health Measurement INstruments) defined criteria for the selection of PROMs for COS, which include high-quality evidence for feasibility, good content validity, and good internal consistency [100].

A common approach for clarifying which tools can be used to measure distinct health outcomes is to link their contents to the ICF [101,102]. Gilrichst et al. provide a list of numerous COAs for the evaluation of different components of the ICF, which could be used in cancer rehabilitation [103]. They give a comprehensive overview of available instruments, but do not compare their psychometric qualities. This would be a necessary next step to inform the selection of instruments.

The International Consortium for Health Outcomes Measurement (ICHOM) has developed so-called Standard Sets (ICHOM-SS) for outcome assessments in various fields, including oncology [104,105,106,107,108]. They are based on a concise methodology involving researchers and patients. Within a consensus process, not only essential outcome domains but also optimal measurement time-points, risk-adjustments, and important baseline variables (demographical data and health status) are discussed. Not all, but a considerable portion of the outcomes of interest can be assessed by PROMs. The EORTC QLQ-C30 is recommended in nearly all cancer-specific ICHOM-SS, including standard sets for breast, lung, colorectal, and advanced prostate cancer [104,106,107,108].

As briefly described above, the EORTC QLQ-C30 is a very useful instrument, as it covers several domains of HRQOL that are known to be relevant for patients with cancer, while only including 30 items. At the same time, it can be supplemented by various more specific modules if necessary. The EORTC QLQ-C30 is the most frequently used questionnaire in clinical trials [109] and extensive normative data exist [110,111,112], which allow comparisons between different populations. Additionally, the EORTC survivorship questionnaires allow the more specific assessment of cancer survivors’ HRQOL [66,72], although they do not (yet) offer the same compatibility as the EORTC QLQ-C30 as the measures are still only newly developed.

To conclude, several recommendations offer guidance for the selection of PROMs for use in cancer rehabilitation settings. In general, it is advised to use well-validated instruments such as the EORTC QLQ-C30 and its modules. Besides the EORTC QLQ-C30 and its modules (https://qol.eortc.org/, accessed on 15 December 2021), the FACIT measures (https://www.facit.org/, accessed on 15 December 2021) or the cancer-specific PROMIS scales (https://www.healthmeasures.net/, accessed on 15 December 2021) are widespread and well validated. The selection of instruments for different studies depends on the specific target population and the purpose of the study. The tool of choice must be valid and reliable for use in the specific study context.

## 7. Integration of (Electronic) PROs into the Rehabilitation Setting

The next step after the selection of PROMs is to implement them in the cancer rehabilitation setting. This is a critical step, as even the most carefully selected and best-validated PROMs will not be useful in practice if they are poorly integrated into clinical care. In the past years, several landmark studies were able to show the potential benefits of well-implemented PROs for patient care, especially during treatment: the use of PRO monitoring has been shown to significantly prolong overall survival [113,114] and to decrease hospitalizations [115]. There are also studies on how PROs can supplement daily clinical care, enrich patient–clinician communication, help identify relevant symptoms, and increase HRQOL [116,117,118,119,120,121]. Even though such studies have mainly been conducted in populations receiving active treatment and evidence in the post-treatment setting is more scarce [122], it is still likely that cancer rehabilitation may profit from similar effects. While effects on overall survival are improbable, the focus of rehabilitation on restoring functioning and HRQOL is in harmony with the use of PROMs.

Increasing efforts are being undertaken to enable electronic PRO collection (ePROs), which offer several advantages. Compared to paper-pencil assessments, ePROs offer higher data quality, as they produce fewer missing answers [123], while offering the same validity as traditional paper questionnaires [124]. Even more importantly, ePROs allow automated and instantaneous scoring and graphical presentation of PROMs, which is a prerequisite for effective use in clinical practice [125]. Via a link to the provider’s electronic health record (EHR), ePROs can be integrated into the electronic care pathway. Finally, ePROs offer many additional benefits, such as automated reminders to complete questionnaires, which can increase patient engagement [126,127]. This also supports post-rehabilitation follow-up assessments, which can be used to evaluate the long-term effects of rehabilitation.

Of course, electronic systems can be used not only to record ePROs, but also other types of COAs, such as ClinROs. The clinical value of an electronic system increases if it is used by patients and clinicians alike and if it integrates and combines different sources of data. As this review is focused on PROs specifically, we will further elaborate on ePROs in this chapter.

By now, there are many different software providers that offer solutions for ePRO integration and patient platforms offering multiple features (for a review of systems, see [128]). However, the difficulty in getting ePROs into healthcare is not only a technical one. The crucial part is tailoring the ePRO system to the given situation and application [129,130,131]. For example, the clinical value of an electronic system increases if it is used by patients and clinicians alike and if it integrates and combines different sources of data (e.g., electronic health record, PROs, ClinROs, and PerfO results). There is no one-size-fits-all solution to implementations (similar to the selection of PROMs), which makes implementation on a large scale difficult. If PROMs are not sufficiently integrated into and tailored toward the rehabilitation process, providers and clinicians will not see their benefits and usage will stagnate.

The implementation of (e)PROs in the healthcare process can be considered a complex intervention that affects the provision of care on many levels [129]. Considering the roles of different stakeholders (e.g., administrative personnel, nurses, and physicians) in the implementation and engaging them in the process is advisable [130,132]. Aside from healthcare professionals’ motivation and use of the systems, the role and motivation of the patient is central. As noted by Basch et al., “Unlike the other care enhancements, implementation of patient-reported outcomes requires participation by the patient. For a patient-reported outcomes program to succeed, patients must be successfully and durably engaged” [83]. Vulnerable patients in particular (older, less educated, and worse HRQOL) are known to participate less frequently in PRO follow-up assessments, which can introduce bias in the analysis of data [98]. However, there is also evidence that even older patients, such as older cancer survivors, are able to complete ePRO assessments reliably [133] and are open, regardless of age, to using information health technology to communicate with their care team [134].

Consequently, any implementation should be carefully planned and executed. Detailed instructions on how to implement (e)PROs in cancer rehabilitation are beyond the scope of this review, but different guidance documents and recommendations exist. The International Society for Quality of Life Research (ISOQOL) [135] and the EORTC have published guides to facilitate the implementation of PROMs in daily clinical practice [131]. In general, following an implementation science approach is advisable to account for the complexity of implementations [129]. As a concrete example of supporting cancer rehabilitation, Wintner et al. described the process of implementing an ePRO monitoring system called CHES (Computer-based Health Evaluation System [136]) in an Austrian healthcare center [130]. In their paper, Wintner et al. discussed the challenges that arose and provide advice on the different steps in the implementation process.

### 7.1. Barriers to Successful Implementation

There are several potential barriers to the integration of ePROs into clinical care. Notably, while the drivers of successful implementations vary for different settings (i.e., different settings may have different drivers of change), the barriers are often similar [129]. Some of the most common barriers ePRO implementations face are already-full workflows of clinical staff and a lack of administrative resources, unclear interpretation of the PROM results on the part of both clinicians and patients, and a lack of clinician usage of the PROM data [135,137,138,139]. The latter is a crucial point, as PROMs have the potential to enhance clinical care by informing treatment decisions [140,141], supporting patient empowerment [142], and shared decision making [141,143,144], all of which rely on stakeholder buy-in and clinical use of PROMs.

There are also barriers in information technology and ePRO systems. Not all commonly used EHR systems have integrated features to meaningfully present PROM data and many lack supporting information on or help for interpreting the data [145,146]. Therefore, electronic systems are required that support not only healthcare professionals, but also patients, in viewing and understanding PROM data [128,136,147,148].

### 7.2. Integrating ePROs into the Rehabilitation Pathway

Another important consideration is how to incorporate ePROs into the rehabilitation pathway. Especially in in-patient rehabilitation, patients often follow a pre-planned schedule into which ePROs can be incorporated. For example, ePROs can be used to optimize referral to therapy or specialists: in the implementation described by Wintner et al., patients who reported higher emotional distress in an ePRO assessment prior to starting the rehabilitation were upfront assigned more psycho-oncological support during their stay [130]. This is just one example of how ePROs can be integrated in post-treatment cancer care. Warrington et al. described potential integrated care pathways for cancer survivors, but also note that further research is necessary before PROMs will be integrated on a broad scale [149].

Due to the great variety of rehabilitation settings and programs, it is difficult to give universal guidance at which time points and how frequently PROs should be assessed. However, the EORTC and ISOQOL offer suggestions on how to decide about time points and frequency of PRO assessments [131,135]. The decision will depend on resources available and might be informed by specific events (e.g., admission, transition to ambulatory settings, outpatient visits, end of rehabilitation, and follow-up) or follow a predefined schedule (e.g., every week/month).

## 8. Conclusions

Our review describes the importance of PROs as the key COA for cancer rehabilitation. PROs are central to assessing the patient perspective on physical, psychological, and social functioning and health-related quality of life. Consequently, they should play a central role in the evaluation of all aspects addressed in comprehensive and holistic cancer rehabilitation care. In this way, PROs can be used to benchmark cancer rehabilitation. Current trends towards value-based care will likely further drive the use of PROs in the future.

However, PROs can only provide a solid basis for decision-making if they are selected carefully, implemented properly, and analyzed correctly. This means that healthcare providers and professionals need to become more acquainted with using PROs. At the moment, there is still a lack of standardization for which PROs should be used, and their implementation in cancer care is highly heterogeneous. Therefore, we recommend that available and upcoming guidance and published policies are followed in order to harmonize the use and analysis of PROs.

Our review paves the way for concrete PRO use in cancer rehabilitation, as we offer advice on which PROMs are available, how to select the right PROMs, and how to implement them using electronic systems. If validated instruments are thoughtfully implemented on a large scale, it will enable us to answer crucial questions on cancer rehabilitation: PROs can be used to evaluate the effectiveness of different kinds of rehabilitation or to identify important predictors for rehabilitation outcomes. More research in these areas will not only provide more sound evidence on why cancer rehabilitation is important, but will also promote more patient-centered care and increase value for patients.
